# Global Prevalence of Childhood Exposure to Physical Violence within Domestic and Family Relationships in the General Population: A Systematic Review and Proportional Meta-Analysis

**DOI:** 10.1177/15248380231179133

**Published:** 2023-06-10

**Authors:** Tyson Whitten, Stacy Tzoumakis, Melissa J. Green, Kimberlie Dean

**Affiliations:** 1 Center for Law and Justice, Charles Sturt University, Port Macquarie, NSW, Australia; 2 Discipline of Psychiatry and Mental Health, University of New South Wales, Kensington, NSW, Australia; 3 School of Criminology and Criminal Justice, Griffith University, Southport, Queensland, Australia; 4 Griffith Criminology Institute, Griffith University, Mount Gravatt, QLD, Australia; 5 Neuroscience Research Australia, Randwick, NSW, Australia; 6 Justice Health and Forensic Mental Health Network, Matraville, NSW, Australia

**Keywords:** family violence, domestic violence, intimate partner violence, childhood trauma, prevalence

## Abstract

Efforts to identify and prevent childhood exposure to physical violence within domestic and family relationships must be underpinned by reliable prevalence estimates to ensure the appropriate allocation of resources and benchmarks for assessing intervention efficacy. We conducted a systematic review and meta-analysis of the global prevalence of childhood exposure to physical domestic and family violence separately as a victim or witness. Searches were conducted in *Criminal Justice Abstracts, Embase, Scopus, PubMed*, *PsychInfo*, and *Google Scholar.* Studies were included if they were peer-reviewed, published in English, had a representative sample, unweighted estimates, and were published between January 2010 and December 2022. One-hundred-and-sixteen studies comprising 56 independent samples were retained. Proportional meta-analysis was conducted to calculate the pooled prevalence for each exposure. Pooled prevalence estimates were also stratified by region and sex. The global pooled prevalence of childhood exposure to physical domestic and family violence as a victim or witness was 17.3% and 16.5%, respectively. Prevalence estimates were highest in West Asia and Africa (victim = 42.8%; witness = 38.3%) and lowest for the Developed Asia Pacific region (victim = 3.7%; witness = 5.4%). Males were 25% more likely than females to be the victim of physical domestic and family violence during childhood, while both were equally likely to have witnessed it. These findings suggest that childhood exposure to domestic and family violence is relatively common, affecting around one-in-six people by 18 years of age globally. Regional variations in prevalence estimates may reflect underlying economic conditions, cultural norms, and service availability.

## Introduction

Physical abuse is the most visible form of domestic and family violence, and is broadly defined to include a range of violent and threatening behaviors within intimate partner and family relationships (Murray & Powell, 2009; Nancarrow et al., 2020). Children who experience physical violence within domestic and family settings, either as a victim (i.e. direct target of actual or threatened violence) or witness (i.e. aware of actual or threatened violence against others), have a significantly greater risk of negative life outcomes, including serious physical and mental illness, low educational attainment, chronic unemployment, and criminal offending, including as a perpetrator of family violence (Artz et al., 2014; Holt et al., 2008; Kimber et al., 2018; Kitzmann et al., 2003; Strathearn et al., 2020; Whitten et al., 2022). Childhood exposure to physical domestic and family violence is particularly pernicious because such acts tend to be covert, frequent, and normalized by the child (Eliffe et al., 2020; Ruddle et al., 2017). The economic burden of this exposure is substantial, costing between 2% and 8% of the global gross domestic product, with annual costs (adjusted for 2022 U.S. dollars) estimated to be up to $3.36 billion in low-middle income countries and $1.20 billion in high-income countries (Pereznieto et al., 2014).

International welfare organizations, governments, and concerned scholars have emphasized the need for concerted commitments to reduce childhood exposure to domestic and family violence as well as providing suitable services and early intervention to those who experience it (Clark et al., 2020; Lee et al., 2016). Such efforts must be underpinned by reliable estimates of the number of children in the general population who experience physical violence within domestic and family settings to: (a) establish its burden and priority for prevention and policy; (b) ascertain baseline prevalence to assess the efficacy of interventions, and; (c) provide the necessary parameters to examine the potential economic impact of an intervention before its implementation (Butchart et al., 2006; Harder, 2014). Consistent with the principles of evidence-based practice, stakeholders should use evidence obtained from systematic reviews and meta-analyses to maximize the quality of the prevalence estimates guiding policy and interventions (Munn et al., 2014; Paul & Leibovici, 2014). This is important for ensuring that intervention and prevention services are appropriately resourced, models assessing program efficacy are underpinned by accurate assumptions, and changes in the prevalence of exposure can be monitored over time.

Prior meta-analyses suggest the global pooled prevalence of childhood physical abuse is 22.6%, with the highest proportions found in Africa (18.9%–60.2%) and the lowest in the Developed Asia Pacific region (6.7%–16.7%) (Mathews et al., 2020; Moody et al., 2018; Stoltenborgh et al., 2015). Global prevalence estimates are also similar for boys and girls, although, when examined separately by region, rates appear to be higher for boys in Africa and Europe (Moody et al., 2018). Nonetheless, these prior estimates were pooled from studies that included physical abuse perpetrated by non-family members (including peers and strangers) and were often sourced from non-representative samples. In addition, children who have *witnessed* physical domestic and family violence have been excluded from previous pooled estimates. However, evidence from individual studies suggest that girls are more likely than boys to witness physical violence between family and household members (Dodaj, 2020).

To date, 196 countries have ratified the United Nations Convention on the Rights of the Child (1989), agreeing to take all appropriate legislative, administrative, social, and educational measures to protect children from violence. Further considerations have also been afforded to reducing violence against women and girls (UN General Assembly, 1993), given they have a higher lifetime risk of domestic violence victimization (Garcia-Moreno et al., 2006). However, there are no population-based benchmarks available to assess if these commitments have had a discernible effect on the global, country, and sex-specific prevalence of children exposed to physical violence within domestic and family settings.

The current study presents a systematic review with meta-analysis of observational studies of international representative samples reporting the prevalence of childhood exposure to physical domestic and family violence separately as a victim or witness. We calculated the pooled prevalence for each exposure, and separately by study region and sex. For this review, childhood exposure to physical domestic and family violence as a victim was defined as being the direct recipient of actual or threatened physical violence by a family member (e.g. parent, grandparent, and sibling), current or former intimate partner of a family member, non-family caregiver (e.g. foster parent), or household member (e.g. foster sibling). Childhood exposure as a witness of domestic and family violence was defined as directly seeing, hearing, or being aware of the immediate consequences of actual or threatened physical violence involving family members, current or former intimate partners of family members, non-family caregivers, or household members. We also use the term “childhood” to refer to the period from age 0 to 18 years (UN General Assembly, 1989).

## Methods

### Search Strategy

The electronic databases and search engines *Criminal Justice Abstracts, Embase, Scopus, PubMed*, *PsychInfo*, and *Google Scholar* were used to search for studies written in English and published in a peer-reviewed journal between January 1, 2010, to December 31, 2022. Studies published before 2010 were not included because we were interested in recent data given ongoing cultural shifts in the norms and perceptions regarding domestic and family violence (e.g. see: Lansford et al., 2017; Webster et al., 2019). Searches were performed on March 10, 2023, and were conducted by flagging the following key terms present in study titles or abstracts: (adverse childhood experience* or ACE*) or ([*domestic* or *interpersonal* or intrafamil* or *family* or *intimate* or partner* or parent* or spouse* or sibling* or brother* or sister* or caregiver*] and [physical abus* or aggress* or *child maltreatment* or *assault* or violen*]) and (child* or adolescen* or *young* or youth* or juvenile*) and (victim* or witness* or expos* or experience* or surviv*) and (*incidence* or *prevalence* or *proportion*). Manual searches of the reference lists of included studies and bibliographies of relevant systematic literature reviews and meta-analyses were also undertaken (Hillis et al., 2016; Hovdestad et al., 2015; Mathews et al., 2020; Moody et al., 2018; Pace et al., 2022; Stoltenborgh et al., 2015). The search strategy was reviewed by a research librarian at the first author’s host institution.

### Eligibility Criteria and Methodological Quality Assessment

Studies were deemed eligible if they met the following criteria: (a) reported observational data from samples representative of the general population (exclude specialized/clinical samples); (b) written in English and published in a peer-reviewed journal between January 1, 2010 to December 31, 2022; (c) reported the *unweighted* period prevalence of respondents who experienced physical domestic and family violence either as a victim or witness across the entire period from 0 to 18 years of age; (d) exposure to physical domestic and family violence as a victim was operationalized as the experience of actual or threatened acts of physical violence by a parent, family member, non-family caregiver, or other household member, any time before the age of 18 years; (e) exposure to physical domestic and family violence as a witness was operationalized as being aware of a parent, family member, non-family caregiver, or anyone else living in the household experience actual or threatened acts of physical violence by a current or former intimate partner, family member, non-family caregiver, or other household member, any time before the age of 18 years.

We used the Joanna Briggs Institute (JBI; Joanna Briggs institute, 2014) Prevalence Critical Appraisal Tool (Munn et al., 2015) to screen out studies of low methodological quality that may bias the findings. This tool assesses the external and internal validity of prevalence data through the following nine questions, some of which we slightly altered to be more specific to this review: (a) *was the sample frame appropriate to address the target population;* (b) *were study participants sampled in an appropriate way;* (c) *was the derived sample size greater than n* *=* *340*^1^; (d) *was the age, sex, and ethnicity of the sample described in detail;* (e) *was the distribution of age, sex, and ethnicity in the study sample relatively proportionate to the expected distribution of the target population;* (f) *was physical domestic and family violence exposure identified using administrative records or validated self-report scales (excluding single item measures);* (g) *was physical domestic and family violence exposure measured in a standard, reliable way for all participants;* (h) *were the numerator and denominator or unweighted proportion of physical domestic and family violence exposure reported, and;* (i) *was the response rate greater than 60%, the attrition rate less than 20%, and missing data less than 10%, and if not, were analyses conducted demonstrating that the absent data did not bias the study*. Responses to each question were coded as 1 (yes) or 0 (no), with scores summed to reflect the overall quality of the study reporting prevalence data. We only included studies that scored positively for questions one, two, seven, and eight, and had an overall score of six or higher (Glasgow et al., 2020; Martins et al., 2019), in addition to meeting our eligibility criteria.

### Study Selection Process

**Figure 1.**
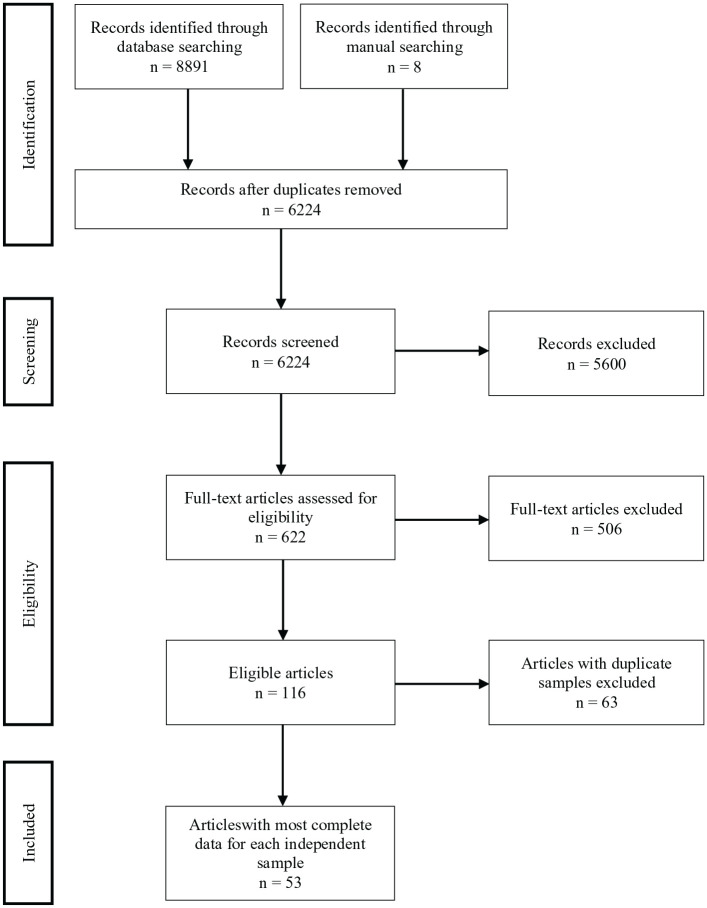
Flowchart of study selection process.

The database search strategy (see Figure 1) identified 8,891 studies, 6,224 of which were unique. Eight additional studies were found through manual searches. All records were exported to Covidence (www.covidence.org) for title, abstract, and full-text screening. After removing duplicates, the titles and abstracts of each study were reviewed for eligibility; 5,600 studies were deemed ineligible. The full texts of the remaining 622 studies were reviewed. Over half (*n* = 342) of the studies were excluded because of unrepresentative samples, 129 were excluded because of ineligible measures of childhood exposure to physical domestic and family violence, and 25 were excluded because the sample included participants under the age of 18 years. The remaining 126 studies initially met our inclusion criteria, although 10 of these were excluded because they did not meet the threshold for methodological quality. One-hundred-and-sixteen eligible studies comprising 56 independent samples were retained. Where there were multiple studies utilizing the same sample, the studies with the most complete data for each independent sample were included in quantitative analysis to ensure the independence of samples and the inclusion of every participant only once in the relevant meta-analysis. Each step of the screening process was initially conducted by one reviewer (T.W), and then a random 20% of studies at each step were re-inspected by two reviewers (T.W and S.T). The inter-rater agreement at the screening (Cohen’s *k* = 0.94) and data extraction steps (Cohen’s *k* = 1.00) were high. All disagreements about study inclusion were resolved by consensus with the other co-authors (M.J.G and K.D). A flowchart detailing the study selection process is detailed below (Figure 1).

### Data Extraction

A standardized form was created to record all relevant information from the included studies. This included the data source, sampling strategy, study population, sample size, measure(s) of physical domestic and family violence exposure, and prevalence of physical domestic and family violence exposure. Data for each of the outcomes of interest were extracted separately, meaning a single study could provide data for more than one outcome. Data were extracted by one reviewer (T.W.) and a random 20% of studies were independently checked for accuracy by a second reviewer (S.T.)

### Statistical Analysis

Proportional meta-analysis was conducted to calculate the pooled prevalence of childhood exposure to physical domestic and family violence as a (a) victim and (b) witness, separately. Each exposure was treated as a dichotomous variable (no exposure vs. any exposure). The proportions from studies that reported the frequency of exposure based on a Likert response (e.g. 1 = never, 2 = rarely, 3 = sometimes, 4 = often) were dichotomized to indicate any exposure (e.g. 0 = never, 1 = rarely or more). Pooled proportions and 95% CI were obtained using the Freeman-Tukey transformation (arcsine square root transformation) (Newcombe, 1998), which transforms estimates to approximate the Gaussian distribution, reduces variance instability between studies, and is the preferred method of transformation for proportional meta-analysis (Barker et al., 2021; Lin & Xu, 2020; Munn et al., 2015). Weighted summary prevalence estimates were calculated using random effects models, which allow for between-study heterogeneity (e.g. demographic and regional differences) by assuming that individual study proportions follow a normal distribution (Munn et al., 2015).

Statistical heterogeneity was determined by an I^2^ statistic greater than 50% and a significant (*p* > .05) chi square test, indicating that variability in effect estimates were a result of study heterogeneity rather than sampling error (Higgins et al., 2003). Prevalence estimates are assumed to have high heterogeneity due to differences in the time and place studies were conducted, although in the context of proportional meta-analyses this does not necessarily indicate inconsistencies across data (Barker et al., 2021). However, to aid interpretation, we also present the 95% Prediction Intervals (95% PI), which reflect the expected range of the true prevalence in similar studies (Barker et al., 2021; Migliavaca et al., 2022).

Due to the expected heterogeneity between studies, a series of sensitivity analyses examined the difference in pooled prevalence estimates across two potential moderator variables: region and sex (male and female). Regions were categorized according to socioeconomic similarities indicated by the United Nations Department of Economic and Social Affairs (2022), and designated as Developed Asia Pacific (Australia, New Zealand, and Japan), East Asia (China), Europe (United Kingdom, Germany, Poland, Norway, Macedonia, Hungary, and Sweden), North America (United States of America and Canada), South Asia and America (Guyana, Sri Lanka, Cambodia, Papua New Guinea, and Philippines), and West Asia and Africa (India, Saudi Arabia, South Africa, Nigeria, Turkey, Uganda, and Zimbabwe). Comparative meta-analysis between sexes was conducted using the Inverse Variance method to calculate the Odds Ratio (OR) and accompanying 95% CI. Random effect models were used for all analyses. Meta-analyses and forest plots were calculated using JBI SUMARI online software (https://sumari.jbi.global/; Joanna Briggs Institute, 2014; Munn et al., 2019).

## Results

### Summary of Included Studies

Fifty-three studies comprising 56 independent samples were included in the proportional meta-analysis (see Table 1). A considerable proportion of the included studies were based on independent samples from North America (*n* = 17). Independent samples were also often sourced from Europe (*n* = 14), followed by West Asia and Africa (*n* = 7), the Developed Asia Pacific (*n* = 7), East Asia (*n* = 6), and South Asia and America (*n* = 5). Four studies measured physical domestic and family violence exposure prospectively (Doidge et al., 2017; Naicker et al., 2017; Najman et al., 2020; Lacey et al., 2022); all others relied on retrospective self-reports. Half of the studies measured exposure to physical domestic and family violence, either as a victim or witness, using a single item asking respondents if they had experienced the outcome of interest at least once. Two studies used multiple respondents to derive incidents of domestic and family violence exposure (Naicker et al., 2017; Lacey et al., 2022). Only a single study measured physical domestic and family violence exposure using administrative records at the population-level (Rouland et al., 2019).

**Table 1.** Details of Included Studies.

Study Id	Data Source	Study Sample	Measure	% Victim	% Witness
Naiker et al. (2020)	South African Birth to Twenty Plus (BT20+): birth cohort (*N* = 3,270, 51.4% female) born in 1989/90 in Soweto-Johannesburg, South Africa.	*n* = 1,595 at 21/22 year follow up.	Caregiver and participant prospective reports adapted from the Adverse Childhood Experiences International Questionnaire (ACE-IQ) (multi-item indicator).	All = 48.8%	All = 54.2%
Satinsky et al. (2017)	Community-based random household survey of all adults (*N* = 1,795) residing in rural Nyakabre Parish, Uganda, between 2016 and 2018.	*n* = 1,626 (55.8% female), median age 37 years (IQR 26–50).	Modified version of the ACE-IQ (multi-item indicator).	All = 50.7% Male = 50.4%; Female = 50.9%	All = 31.9% Male = 32.6%; Female = 31.3%
Salawu and Owoaje (2020)	Community-based random sample of 575 youths aged 18–35 years residing in Oyo State, Nigeria, in 2013.	*n* = 575 (40.5% female), mean age 26.3 years (SD = 4.9).	Modified version of the ACE-IQ (multi-item indicator).	All = 36.5% Male = 34.2%; Female = 39.9%	All = 23.1% Male = 20.2%; Female = 31.3%
Najman et al. (2020)	Mater University Study of Pregnancy: birth cohort of all infants (*N* = 7,223) born in 1981–1983 at a large metropolitan hospital in Brisbane, Australia.	*n* = 2,417 (57.4% female) at 30 year follow up.	Child Trauma Questionnaire (CTQ) (multi-item indicator).	All = 8.2%	—
Doidge et al. (2017)	Australian Temperament Project: birth cohort of randomly selected infants (*N* = 2,443) who attended an Infant Welfare Centre in 1983 across Victoria, Australia.	*n* = 1,048 (48.1% female) with non-missing data at 23–24 year follow up.	Retrospective report of physical abuse (multi-item indicator) and witnessing domestic violence (single item indicator).	All = 13.5%	All = 11.2%
Fuller-Thomson et al. (2013)	2005 Canadian Community Health Survey: cross-sectional population-based survey of around 130,000 Canadians aged 12 years or older residing in private dwellings.	*n* = 13,054 (56.1% female) regional subsample aged 18 years or older, with 54.9% aged 30–59 years.	Retrospective report of physical abuse (single item indicator).	All = 7.8% Male = 4.8% Female = 10.2%	—
Shields et al. (2016)	2012 Canadian Community Health Survey—Mental Health: cross-sectional population-based of 25,113 Canadians aged 15 years or older residing in private dwellings.	*n* = 21,878 over 18 years of age.	Childhood Experiences of Violence Questionnaire (CEVQ) (multi-item indicator).	All = 25.8%	All = 8.1%
Fu and Chen (2022)	Community-based random sample of 2,061 elders (65 years or older) originally surveyed in 2018 in Beijing, China in 2018.	*n* = 1002 (53.6% female) followed up in 2019, mean age 74.9 years (SD = 6.2).	Retrospective report of physical abuse (single item) and witnessing domestic violence (single item indicator).	All = 7.9%	All = 7.7%
Chan et al. (2021)	Random household survey of 8,945 adults residing in six cities across China in 2009/10.	*n* = 8807 (56.6% female), mean age 40.6 years (SD = 8.9).	Modified Chinese version of the revised Conflict Tactics Scale (CTS2) (multi-item indicator).	All = 8.3%	All = 3.8%
Cheng et al. (2011)	World Mental Health Surveys—metropolitan China Initiative: Random household survey of adults residing in Beijing and Shanghai, China.	*n* = 1611 aged 18–70 years.	Chinese version of the World Mental Health Composite International Diagnostic Interview (WMH-CIDI) (multi-item indicator).	All = 14.6%	—
Wang (2020)	China Health and Retirement Longitudinal Study (CHARLS): longitudinal, population-based sample of 18,780 adults residing in China in 2011.	*n* = 15, 450 (51.7% female) followed up in 2015, with mean age 59.5 years (SD = 9.9).	Retrospective report of physical abuse (single item indicator).	All = 28.6% Male = 34.3% Female = 23.3%	—
Ulke et al. (2021)	Community-based random household survey of 13,964 participants aged above 13 years in Berlin, Germany, in 2010, 2013, and 2016.	*n* = 5,836 (53.9% female) born before 1981, median age 56 years.	German version of the Childhood Trauma Screener (CTS) (single item indicator).	All = 18.1% Male = 20.2% female = 16.3%	—
Klinger et al. (2022)	German National Cohort (NAKO): population-based study of 205,000 random participants across Germany between 2014 and 2019.	*n* = 76, 731 (52.7% female), mean age 51.4 years (SD = 12.1).	German version of the CTS (single item indicator).	All = 20.1%	—
Häuser et al. (2019)	Population-based random household survey of 2,508 participants aged 14 years or older across Germany in 2013.	*n* = 2,416 (53.5% female) aged 18 years or older, mean age 50.8 years (SD = 17.5).	German version of the CTS (single item indicator).	All = 27.7%	—
Nagy et al. (2019)	Random household survey of 1,200 adults across Hungary in 2016.	*n* = 1,174 (63.5% female), mean age 53.2 years (SD = 16.5).	Adverse Childhood Experiences (ACE) Score Calculator (multi-item indicator).	All = 5.0% Male = 4.1% Female = 6.0%	All = 5.0% Male = 6.1% Female = 4.0%
Fernandes et al. (2021)	Consortium on Vulnerability to Externalizing Disorders and Addiction: community-based sample of 6,120 (52.2% Female) participants aged 13–23 years across six catchment areas in India in 2016.	*n* = 2,751 aged 18–23 years.	Modified version of the ACE-IQ (multi-item indicator).	All = 28.4%	All = 34.0%
Yanagi et al. (2020)	Japan Gerontological Evaluation Study: population-based study of 137,735 participants (ACEs investigated in subsample of *n* = 25,928) aged 65 years or older across 15 prefectures in Japan in 2013.	*n* = 24,271 (52.9% female) with non-missing data, mean age 73.2 years (SD = 5.9).	Retrospective report of physical abuse (single item indicator) and witnessing domestic violence (single item indicator).	All = 1.3% Male = 1.9% Female = 0.7%	All = 3.6% Male = 4.3% Female = 3.1%
Tsuboi et al. (2015)	Lifestyle and Attitudes Toward Sexual Behavior survey: population-based study of 3,000 participants aged 16–49 years residing in Japan in 2010.	*n* = 1,414 (56.9% female) aged 20–49 years with non-missing data.	Retrospective report of physical abuse (single item indicator).	All = 2.9% Male = 1.8% Female = 3.7%	—
Raleva (2018)	Stratified random sample of 1,277 youth (18–21 years) residing in the Republic of Macedonia.	*n* = 1,277 (58.6% female), mean age 20.0 years (SD = 2.73).	Modified version of Family Health History questionnaires (multi-item indicator).	All = 21.1% Male = 22.3% Female = = 20.2%	All = 10.1% Male = 9.1% Female = 10.8%
Rouland et al. (2019)	New Zealand Integrated Data Infrastructure: Whole-of-population birth cohort of 56,904 children born in New Zealand in 1998.	*n* = 56,904 followed to 18 years of age.	Prospective records from Child Protection Services indicating substantiated maltreatment	All = 3.1%	—
Huang and Mossige (2018)	Norwegian Youth Survey on Violence and Abuse: stratified random sample of 7,033 students attending last year of secondary school in Norway in 2007.	*n* = 7,033 (58.5% female) aged 18–20 years.	Retrospective report of physical abuse (multi-item indicator) and witnessing domestic violence (multi-item indicator).	All = 24.0%	All = 11.8%
Ramiro et al. (2010)	Community-based random household survey of 1,068 adults aged 35 years or older residing in Quezon City, Philippines, in 2007.	*n* = 1,068 (49.9% female), mean age 46.7 years (SD = 9.2)	Modified version of Family Health History questionnaires (multi-item indicator).	All = 1.3% Male = 1.1% Female = 1.5%	All = 17.7% Male = 21.9% Female = 13.5%
Schulz et al. (2014)	Study of Health in Pomerania: Population-based cohort study of 6,267 adults residing in West Pomerania from 1997 to 2001.	*n* = 2,038 with non-missing data at 2007/10 follow up, median age 56 years (range 29–89)	German version of the CTQ (multi-item indicator).	All = 8.7%	—
Almuneef et al. (2018)	Population-based random household survey of 10,000 adult Saudi citizens raised and currently living in Saudi Arabia in 2013.	*n* = 9,533 (48% female), mean age 34 years (SD = 11).	Modified version of the ACE-IQ (multi-item indicator).	All = 44.6%	All = 57.0%
McLafferty et al. (2018)	Northern Ireland Study of Health and Stress: Population-based random household survey of 4,340 Adults (56.4%) residing in Northern Ireland in 2004–2008.	*n* = 1,986 (52.2% female), mean age 45 years.	Modified version of the CTS (single item indicator).	All = 4.2% Male = 4.4% Female = 3.0%	All = 6.2% Male = 5.4% Female = 5.4%
Lacey et al. (2022)	Avon Longitudinal Study of Parents and Children: Birth cohort of 14,775 infants born in Bristol, England, in 1991/92.	*n* = 1302–3377 (48.9% female) with non-missing data at 19 year follow up	Mother and her partners’ prospective reports of physical abuse (multi-item indicator) and witnessing domestic violence (multi-item indicator).	All = 10.4%	All = 9.2%
Bellis et al. (2014)	Random household survey of 3,885 participants aged 18–69 years residing across England in 2013.	*n* = 3,885 (55% female), 40.8% aged 18 to 39 years.	CDC short ACE tool (single item indicator).	All = 14.3% Male = 14.9% Female = 13.8%	All = 12.1% Male = 11.5% Female = 12.6%
Bürgin et al. (2021)	U.S. Health and Retirement Study: longitudinal, population-based sample of 43,478 participants aged over 50 years residing in the U.S. in 1992–2012	*n* = 15,717 (58.3% female) with non-missing data at 2008, 2010, or 2012 follow-up, mean age 67.6 years (SD = 20.6).	Retrospective report of physical abuse (single item indicator).	All = 7.0% Male = 6.1% Female = 7.7%	—
Iverson et al. (2013)	National Comorbidity Survey—Replication Survey: Population-based random household survey of 9,282 adults with a permanent residence in the U.S. between 2001 and 2003.	*n* = 5,692 (53.1% female) with non-missing data, 62.4% aged 18–49 years.	Retrospective report of physical abuse (single item indicator) and witnessing domestic violence (single item indicator).	All = 9.0% Male = 7.7% Female = 10.1%	All = 17.7% Male = 14.4% Female = 20.6%
Fang and McNeil (2017)	2012 Behavioral Risk Factor Surveillance System (BRFSS): annual cross-sectional telephone survey administered to a population-based sample of adults in the U.S. (N = 39,434).	*n* = 25,809 (63.9% female), 33.3% aged 18–49 years	CDC short ACE tool (single item indicator).	All = 14.0%	All = 15.1%
Scheidell et al. (2018)	National Longitudinal Study of Adolescent to Adult Health (Add Health): longitudinal, representative cohort of 20,745 adolescents aged 11–21 years in 1994/95 in the U.S.	*n* = 12,288 (54.4% female), with non-missing data at 24–34 year follow up.	Retrospective report of physical abuse (single item indicator)	All = 12.0%	—
Ford et al. (2011)	2009 BRFSS (*N* = 29,212).	*n* = 25,809 (63.9% female), 33.3% aged 18–49 years.	CDC short ACE tool (single item indicator).	All = 14.0%	All = 15.1%
Alcalá et al. (2016)	2011 BRFSS (*N* = 131,686).	*n* = 111,964 (59.6% female), mean age 46 years	CDC short ACE tool (single item indicator).	All = 15.5%	All = 14.8%
Hazzard et al. (2021)	Eating and Activity over Time: longitudinal, representative cohort of 2,793 adolescents attending school in urban Minnesota, U.S., in 2009/10.	*n* = 1,440 (58.3% female) with non-missing data at 2017/18 follow-up, mean age 22.2 years (SD = 2.0)	Modified version of the CTQ (single item indicator).	All = 16.1%	—
Anderson and Blosnich (2013)	2010 BRFSS (*N* = 22,071)	*n* = 20,713 (60.5% female), mean age 56.5 years (SD = 0.12).	CDC short ACE tool (single item indicator).	All = 16.7%	All = 15.2%
Afifi et al. (2011)	National Epidemiologic Survey on Alcohol and Related Conditions (NESARC): representative sample of 34,653 participants aged 20 years or older residing in the U.S. in 2004/05.	*n* = 34,653 aged 20 years or older.	CTS (single item indicator).	All = 18.2%	—
Afifi et al. (2017)	NESARC wave 3: cross-sectional, representative sample of 36,309 participants aged 18 years or older residing in the U.S. in 2012/13.	*n* = 36,309 (56.3% female), mean age 46.5 years (SD = 0.2).	CTS (single item indicator).	All = 17.9% Male = 18.4% Female = 17.5%	All = 18.5% Male = 16.9% Female = 19.8%
Ozieh et al. (2020)	Midlife Development in the United States: longitudinal telephone survey administered to a population-based sample of 7,108 participants aged 25–74 years residing in the U.S. in 1995/96.	*n* = 1,205 (56.8%) with non-missing data at 2011/16 follow-up, median age 57 years (IQR = 49–65).	Retrospective report of physical abuse (multi-item indicator).	All = 22.2%	—
Wade et al. (2016)	Philadelphia ACE survey: cross-sectional telephone survey administered to a random sample of 1,784 adults residing in Philadelphia, U.S.	*n* = 1,784 (58.3% female), mean age 48.6 years (SD = 0.6).	Retrospective report of physical abuse (multi-item indicator) and witnessing domestic violence (multi-item indicator).	All = 30.8%	All = 16.1%
Baiden et al. (2022)	2019 BRFSS (*N* = 418,268)	*n* = 41,322 (49.3%), 62.2% aged 18–44 years.	CDC short ACE tool (single item indicator).	All = 25.3%	All = 19.0%
Cavanaugh et al. (2015)	NESARC (*N* = 34,653)	*n* = 34,652 (57.8% female), mean age 48.2 years (se = 0.01).	CTS (single item indicator).	—	All = 9.2% Male = 7.5% Female = 10.4%
Cater et al. (2015)	RESUMÈ-project: Population-based sample of 2,500 participants born in 1987–1991 and residing in Sweden in 2011.	*n* = 2,500 (52.6% female) aged 20–24 years.	Modified version of the Childhood Exposure to Domestic Violence Questionnaire (multi-item indicator).	—	All = 28.1% Male = 25.0% Female = 30.9%
Liao et al. (2021)	CHARLS (*N* = 18,780)	*n* = 9,910 (50.8% female) aged 60 years or older.	Retrospective report of physical abuse (single item indicator).	—	All = 14.9%
Radcliff et al. (2018)	2014/15 BRFSS (*N* = 19,843).	*n* = 18,176 (58.7% female), 31.5% aged 18–49 years.	CDC short ACE tool (single item indicator).	—	All = 16.3%
Saraçlı et al. (2016)	Cross-sectional household survey administered to 899 randomly selected participants aged 18–65 years residing in Zonguldak city, Turkey.	*n* = 897 (52.4% female), mean age 39.4 (SD = 12.4 years).	Turkish version of the CTQ (single item indicator).	All = 22.5%	All = 31.0%
Chigiji et al. (2018)	Cross-sectional household survey of 2,410 randomly selected participants aged 13–24 years residing in Zimbabwe, Africa.	*n* = 1,156 (49% female) aged between 18 and 24 years.	Retrospective report of physical abuse (single item indicator).	All = 70.2% Male = 76.1% Female = 63.8%	—
Liang et al. (2022)	UK Biobank: population-based cohort of >500,000 participants aged between 40 and 70 years residing in the UK between 2006 and 2010.	*n* = 145,374 (56.4% female), mean age 55.9 (SD = 7.7) years, who completed online questionnaire.	CTS (single item indicator).	All = 8.0%	—
Peltzer and Pengpid (2022)	World Mental Health Surveys: Multistage household probability sample of 2,662 participants aged 18–69 years in Guyana.	*n* = 2,660 (59.9% female), 69.3% aged 18–44 years.	Retrospective report of physical abuse (single item indicator).	All = 42.1%	—
Morita and Fujiwara (2020)	Population-based cross-sectional household survey of 4,839 residents aged 65 years or older living in Wakuya City, Japan.	*n* = 1,140 (53.4%), mean age 74.7 (SD = 7.1) years, with non-missing data.	Retrospective report of physical abuse (single item indicator) and witnessing domestic violence (single item indicator).	All = 1.5%	All = 4.1%
Fulu et al. (2017)	UN Multi-Country Study on Men and Violence: Population-based household surveys with multistage representative sampling of participants aged 18–49 years in Cambodia, China, Papua New Guinea, and Sri Lanka.	Cambodia *n* = 2,289 (20.8% female). China *n* = 2,101 (52.5% female) Papua New Guinea *n* = 1,737 (50.3%) Sri Lanka *n* = 2,186 (29.9%)	Modified version of the CTQ (multi-item indicator).	Cambodia: All = 45.7% Male = 44.9% Female = 48.6% China: All = 18.0% Male = 25.8% Female = 11.1% Papua New Guinea: All = 57.8% Male = 67.2% Female = 48.5% Sri Lanka: All = 32.1% Male = 37.6% Female = 19.3%	Cambodia: All = 23.3% Male = 23.9% Female = 21.2% China: All = 19.8% Male = 20.9% Female = 18.9% Papua New Guinea: All = 52.5% Male = 56.0% Female = 49.0% Sri Lanka: All = 25.5% Male = 30.3% Female = 14.4%
Koyama et al. (2022)	Neuron to Environmental Impact Across Generations Study: Cross sectional, stratified random sample of 1,346 residents aged 65–84 living in Tokamachi City, Japan.	*n* = 491 (56.6%), mean age 72.8 (SD = 5.5) years with non-missing data.	Japanese version of the ACE-IQ (multi-item indicator).	All = 1.0% Male = 2.8% Female = 0.5%	All = 3.9% Male = 7.1% Female = 4.9%
Riem and Karreman (2019)	Longitudinal Internet Studies for the Social Sciences: Online panel of around 7,500 participants randomly selected from households in the Netherlands.	*n* = 3,586 (52.6% female) aged 18–92 years (*m* = 54.9 years [SD = 14.7]) with non-missing data at waves 1 and 2.	Retrospective report of physical abuse (single item indicator).	All = 3.2%	—
Petersen et al. (2022)	Nationwide representative household survey of 2,519 German residents.	*n* = 2,288 (52.3% female) aged 25 years or older (m = 53.3 [SD = 16.1]) years.	German version of the ACE-IQ (multi-item indicator).	All = 12.6%	All = 6.6%

### Proportional Meta-analysis: Victim

**Figure 2.**
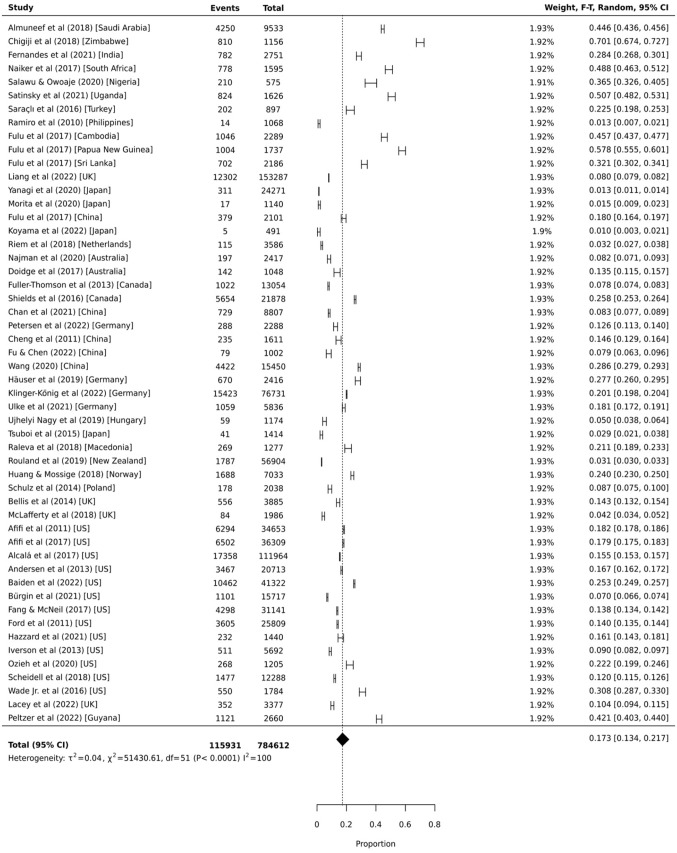
Proportional meta-analysis of childhood exposure to physical domestic and family violence as a victim (*k* = 52).

**Figure 3.**
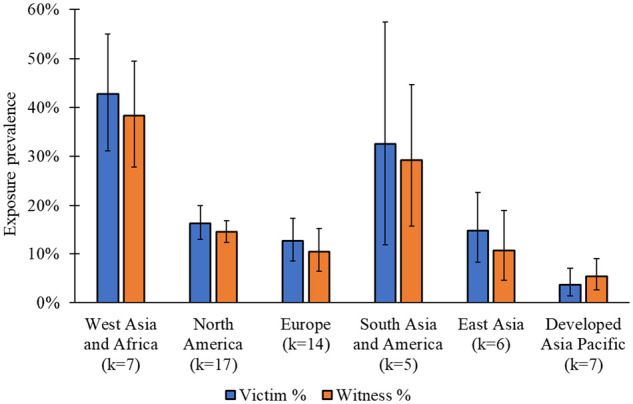
Prevalence of childhood exposure to physical domestic and family violence as a victim and witness by region (*k* = 56).

Fifty-two independent samples provided data on the prevalence of childhood physical domestic and family violence victimization. As presented in Figure 2, the global pooled prevalence estimate was 17.3% (95% CI = 13.4%, 21.7%, 95% PI = 11.3%, 25.5%, *I*^2^ = 99.9%). Individual sample prevalence estimates ranged from 1.0% to 70.1% and appeared to be lowest for Japan (1.0%–2.9%) and highest for Africa (36.5%–70.1%). Stratification by region indicates that almost half of those from West Asia and Africa (*k* = 7; 42.8% [95% CI = 31.1%, 55.0%, 95% PI = 25.5%, 62.1%, *I*^2^ = 99.5%]), and one-third of those from South Asia and America (*k* = 5; 32.5% [95% CI = 11.9%, 57.4%, 95% PI = 6.7%, 76.3%]), experienced physical domestic and family violence victimization by 18 years of age (see Figure 3). A smaller proportion experienced childhood physical domestic and family violence victimization in North America (*k* = 15; 16.3% [95% CI = 13.0%, 19.9%, 95% PI = 12.1%, 21.6%, *I*^2^ = 99.9%]), East Asia (*k* = 5; 14.8% [95% CI = 8.3%, 22.7%, 95% PI = 6.5%, 30.2%, *I*^2^ = 99.6%]), and Europe (*k* = 13; 12.7% [95% CI = 8.6%, 17.4%, 95% PI = 8.3%, 18.9%, *I*^2^ = 99.9%]). The Developed Asia Pacific region had the lowest pooled prevalence of victimization (*k* = 7; 3.7% [95% CI = 1.4%, 7.1%, 95% PI = 1.4%, 8.9%, *I*^2^ = 99.7%]).

**Figure 4.**
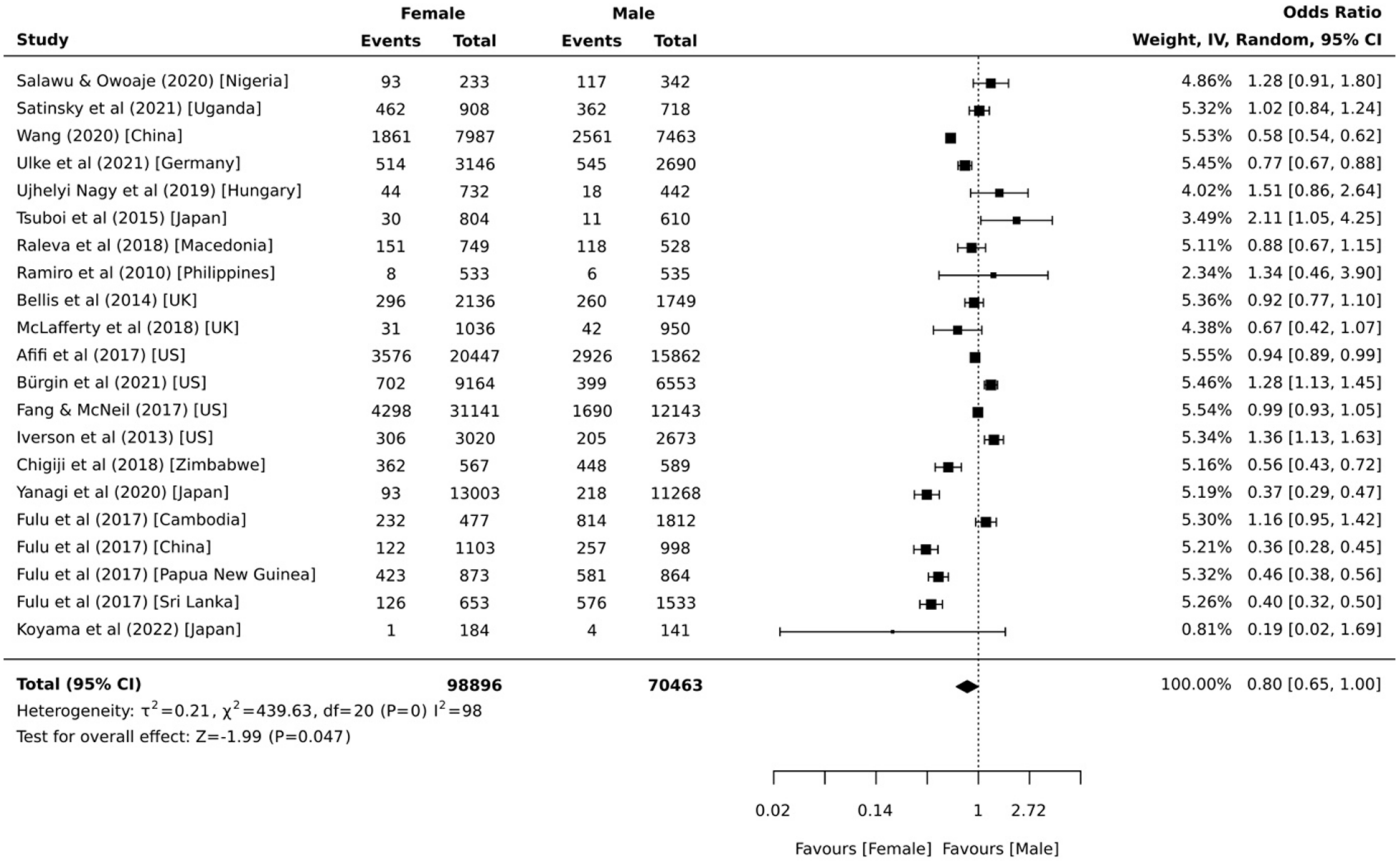
Comparative meta-analysis of childhood exposure to physical domestic and family violence as a victim by sex (*k* = 21).

Data from 21 independent samples provided prevalence estimates stratified by sex. The proportions from the individual samples ranged from 0.5% to 63.8% for females and 1.1% to 76.1% for males. The pooled analyses indicates that 16.7% (95% CI = 9.6%, 25.2%, 95% PI = 8.6%, 29.9%, *I*^2^ = 99.9%) of females and 20.6% (95% CI = 12.2%, 30.6%, 95% PI = 10.5%, 36.4%, *I*^2^ = 99.9%) of males were the victims of physical violence within domestic and family settings by 18 years of age. The sex comparisons presented in Figure 4 show that males were 1.25 (95% CI = 1.00, 1.54, 95% PI = 0.47, 3.35, *I*^2^ = 98.0%) times more likely than females to be childhood victims of physical domestic and family violence. This did not change after removing the three samples with the most unstable estimates (Koyama et al., 2022; Ramiro et al., 2010; Tsuboi et al., 2015) (OR = 1.28 [95% CI = 1.04, 1.59, 95% PI = 0.49, 3.36, *I*^2^ = 98.0%]). Separate analyses by region indicate that males were significantly more likely than females to be childhood victims of physical domestic and family violence in East Asia (*k* = 2; OR = 2.17 [95% CI = 1.35, 3.45, *I*^2^ = 93.0%]) and Europe (*k* = 5; OR = 1.18 [95% CI = 1.02, 1.35, 95% PI = 0.80, 1.74, *I*^2^ = 34.0%]) only.

### Proportional Meta-analysis: Witness

**Figure 5.**
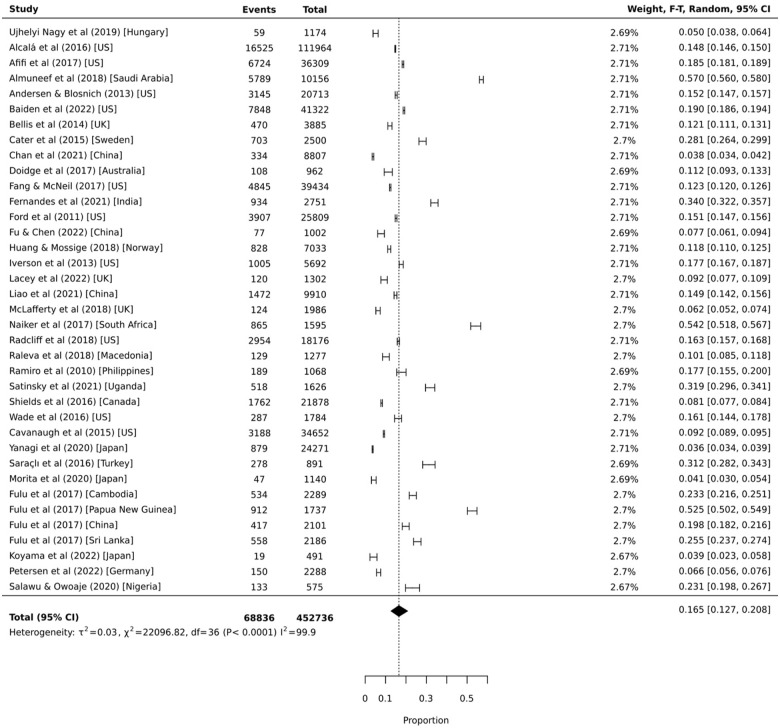
Proportional meta-analysis of childhood exposure to domestic and family violence as a witness (*k* = 37).

Thirty-seven independent samples provided prevalence data on exposure to physical domestic and family violence as a witness. As displayed in Figure 5, the global pooled prevalence was 16.5% (95% CI = 12.7%, 20.8%, 95% PI = 11.1%, 23.8%, *I*^2^ = 99.9%). Prevalence estimates from individual studies ranged from 3.6% to 57.0%. Stratification by region revealed a similar pattern of exposure to physical domestic and family violence victimization (see Figure 3). That is, proportions were highest for West Asia and Africa (*k* = 6; 38.3% [95% CI = 27.7%, 49.5%, 95% PI = 22.6%, 57.0%, *I*^2^ = 99.4%]), followed by South Asia and America (*k* = 4; 29.1% [95% CI = 15.7%, 44.7%, 95% PI = 7.2%, 68.4%, *I*^2^ = 99.5%]), North America (*k* = 11; 14.5% [95% CI = 12.4%, 16.8%, 95% PI = 12.1%, 17.2%, *I*^2^ = 99.7%]), Europe (*k* = 8; 10.5% [95% CI = 6.5%, 15.3%, 95% PI = 5.8%, 18.2%, *I*^2^ = 99.1%]), East Asia (*k* = 4; 10.7% [95% CI = 4.6%, 18.9%, 95% PI = 2.4%, 36.8%, *I*^2^ = 99.6%]); and the Developed Asia Pacific region (*k* = 4; 5.4% [95% CI = 2.6%, 9.0%, 95% PI = 1.7%, 16.0%, *I*^2^ = 96.9%]).

**Figure 6.**
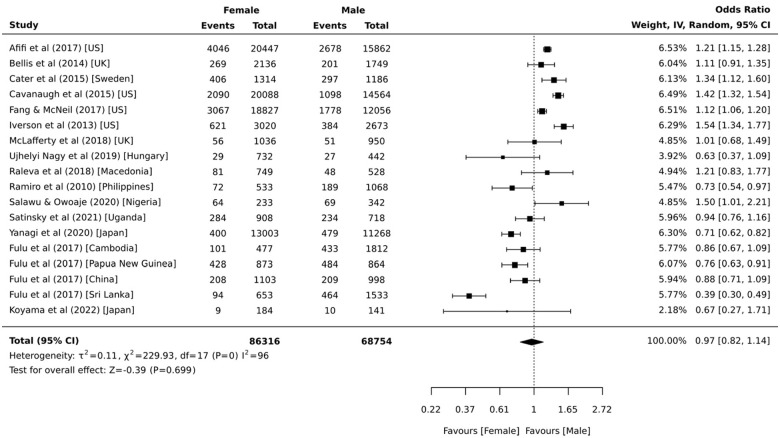
Comparative meta-analysis of childhood exposure to physical domestic and family violence as a witness by sex (*k* = 18).

Data from 18 independent samples provided prevalence estimates stratified by sex. Prevalence estimates ranged from 3.1% to 49.0% for females and 4.3% to 56.0% for males. Pooled prevalence estimates indicate that 16.0% (95% CI = 11.1%, 21.6%, 95% PI = 10.4%, 23.9%, *I*^2^ = 99.7%) of females and 16.6% (95% CI = 11.5%, 22.5%, 95% PI = 10.7%, 24.8%, *I*^2^ = 99.7%) of males witnessed physical violence within domestic and family relationships by 18 years of age. The sex comparisons presented in Figure 6 indicate there was no significant difference between the proportion of females and males who witnessed physical domestic and family violence during childhood (OR = 1.03 [95% CI = 0.88, 1.22, 95% PI = 0.50, 2.13, *I*^2^ = 96.0%]). This did not change when excluding the three samples with the most unstable estimates (Koyama et al., 2022; McLafferty et al., 2018; Ujhelyi Nagy et al., 2019) (OR = 1.01 [95% CI = 0.84, 1.20, 95% PI = 0.47, 2.19, *I*^2^ = 97.0%]). Separate analyses by region indicate that the odds of witnessing physical domestic and family violence during childhood was greater for girls than boys in North America (*k* = 4; OR = 1.31 [95% CI = 1.14, 1.50, 95% PI = 0.67, 2.58, *I*^2^ = 93.0%]) only.

## Discussion

In this study, the pooled prevalence of global exposure to physical domestic and family violence as a victim or witness by 18 years of age was estimated using data from 53 studies comprising of 56 independent samples representative of the general population. Results indicated that, globally, 17.3% of people had been the victim of physical violence by a family or household member, and 16.5% had been a witness. Prevalence estimates were highest for West Asia and Africa, affecting almost half of the pooled population. By contrast, exposure to physical domestic and family violence affected around one-in-eight people in North America, Europe, and East Asia, and only around one-in-twenty people from the Developed Asia Pacific region. Our overall findings suggest that childhood exposure to physical domestic and family violence is relatively common, affecting around one-in-six people by 18 years of age globally, although this varies greatly by region. The multitude of serious adversities following childhood exposure to domestic and family violence warrants that critical steps be taken to reduce its occurrence (Malvaso et al., 2021; Strathearn et al., 2020; Wathen & MacMillan, 2013; Whitten et al., 2022).

The overall prevalence of physical domestic and family violence victimization was lower than those reported in earlier meta-analyses on childhood physical abuse (Stoltenborgh et al., 2015; Moody et al., 2018; Mathews et al., 2020). This is expected, given that we excluded research that measured physical abuse *not* perpetrated by a family or household member, as well as research that did not incorporate representative sampling techniques. Nonetheless, our results conform to the general pattern of findings from prior research indicating that the prevalence of childhood physical abuse tended to be highest for West Asia and Africa, lowest for the Developed Asia Pacific region (Mathews et al., 2020; Moody et al., 2018; Stoltenborgh et al., 2015).

Globally, boys were 25% more likely than girls to be the victim of physical violence within family and domestic settings. Analyses by region indicated that the higher rates of male victimization only occurred in Europe and East Asia. A potential explanation is that corporal punishment by parents is legal in the UK and is used more frequently against boys than girls (Lansford et al., 2010). Furthermore, the social and legal contexts surrounding the one-child policy in China may have led to a higher incidence of physical discipline used against boys due to the greater expectations placed on sons to achieve success, as they are often the parents’ sole heir (Wang & Liu., 2014). By contrast, the global prevalence of witnessing physical domestic and family violence did not differ between boys and girls. However, when stratified by region, girls were 31% more likely than boys to witness family and domestic physical violence in North America. A possible explanation is that girls may have stronger attachments than boys to their mothers, especially in higher socioeconomic countries, and therefore may be more aware of their mothers’ experiences of domestic violence (Dwairy, 2010; Holt et al., 2008; Reynolds et al., 2001).

There was substantial heterogeneity between the reviewed studies. This is not uncommon in proportional meta-analysis (Berker et al., 2021), with similarly large values reported in other studies (for example, see Barth et al., 2013). This is because larger pooled samples increase the I^2^ statistic, independent of clinically relevant variations, as well as true variations across studies in different settings (Rücker et al., 2008). To accommodate this, we calculated prediction intervals to provide a more conservative indicator of uncertainty than confidence intervals (Barker et al., 2021). In proportional meta-analyses, confidence intervals reflect the expected average prevalence of all possible studies, whereas prediction intervals estimate the true prevalence anticipated in future studies across different settings (Higgins et al., 2009). As such, future observations of the prevalence of domestic and family violence have a 95% probability of being between 11.1% and 23.3% for exposure as a victim, and between 8.2% and 26.3% for exposure as a witness, given the results of this study.

### Implications

Few services are available to children who witness domestic and family violence relative to those who are the victim of it, despite both exposures associated with similar negative life consequences (Kitzmann et al., 2003; Holt et al., 2008; Whitten et al., 2022). This, coupled with our finding that an equal proportion of children were the victim or witness of family violence, indicates that all children exposed to domestic and family violence should be afforded the same care as any victim survivor. Similarly, although girls were more likely than boys to have witnessed domestic and family violence, the magnitude of this difference (16%) was small. However, this result was the product of relatively few studies, most of which were from high-income countries from the Northern hemisphere. As such, more research that disaggregates prevalence estimates by sex from a diversity of regions are needed. Furthermore, an equal proportion of boys and girls were also the victim of domestic and family violence, which is in line with previous reviews on physical abuse (Moody et al., 2018; Stoltenborgh et al., 2013).

The prevalence of childhood exposure to physical domestic and family violence was generally highest in lower income countries and may reflect regional variations in cultural norms. For example, family violence appears to be traditionally accepted in some parts of India and Sub-Saharan Africa (Alesina et al., 2021; Kimuna et al., 2012). In other countries, such as Japan, family violence may be underreported because it is culturally perceived to be a private matter that could bring shame to the family (Keen et al., 2015). Moreover, corporal punishment against children by parents is legal in many countries, including most African and Asian nations (The Global Initiative to end All Corporal Punishment of Children, 2019). Corporal punishment is also permissible in many high-income countries, such as the United Kingdom, Canada, and Australia, if it does not amount to visible or lasting injury.

Most countries have ratified the United Nation’s Convention on the Rights of the Child (1989), which stipulated that governments would take all reasonable legislative, administrative, social, and educational measures to protect children from violence. Nonetheless, childhood exposure to physical domestic and family violence appears to still be widespread, particularly in West Asia and Africa. However, efforts to reduce children’s exposure to violence in these countries may be hampered by volatile political and economic conditions that restrict opportunities for cultural and legislative change. As such, governments and international welfare organizations may need to place greater priority on countries that require additional assistance and humanitarian aid in attempts to provide culturally sensitive domestic and family violence prevention and intervention services.

Our findings are at odds with previous data which underpins modeling of the economic burden of childhood exposure to domestic and family violence. For example, Holmes and colleagues (2018) estimated that the average lifetime costs of childhood exposure to domestic and family violence in the general US population was over $50,000 per victim and $55 billion nationwide, based on an estimated exposure prevalence of 25%; a figure derived from a survey of 4,000 children on their experiences of any type of child maltreatment, including both non-family violence (i.e. forms of violence not perpetrated by family members) and family violence (Finkelhor et al., 2015). Our estimates (which did not include non-family violence) suggest that the prevalence of exposure to domestic violence perpetrated by family members in the US is closer to 16%. These discrepancies in estimates of prevalence should be considered in future economic modeling to account for variation in prevalence derived from different types of data, as this has direct implications for the implementation of policy and intervention.

### Limitations and Future Directions

The reliability of our prevalence estimates must be considered within the context of the included studies. Firstly, most studies used retrospective self-reports to measure domestic and family violence exposure. While self-reports may capture instances of domestic and family violence not detected by official records (Stoltenborgh et al., 2015), they are susceptible to recall and response bias (Bauhoff, 2014). Such threats to reliability may be offset by corroborating self-report data from multiple respondents, although this was only done in two of the included studies and the proportions reported were consistent with other studies from the same region. Furthermore, domestic and family violence exposure was often measured by a single question, usually “did your parents or adults in your home ever hit, beat, kick, or physically hurt you in any way (do not include spanking)?” or “did your parents or adults in your home ever slap, hit, kick, punch, or beat each other up?.” A single item cannot capture the complex contexts and nuances associated with domestic and family violence. Finally, although we categorized studies by regions that reflect similar socioeconomic characteristics, the countries included in each category may not reflect similar cultures or values regarding domestic and family violence.

Several methodological limitations should be considered when interpreting these findings. First, there was an overrepresentation of studies from high-income countries with predominantly Caucasian populations, particularly the U.S.A. Therefore, our global prevalence estimates may be biased in favor of this demographic. Second, we excluded behaviors that are recognized as forms of domestic and family violence (e.g. coercive control and emotional manipulation) but do not reflect actual or threatened physical harm. Inclusion of these other behaviors would likely produce higher prevalence estimates. Finally, we were unable to assess the frequency or severity of exposure to domestic and family violence.

Additional research is needed to obtain more reliable estimates of childhood domestic and family violence exposure in the general population. First, more evidence from whole-of-population record-linkage studies are needed, particularly those capable of creating multi-agency indicators of domestic and family violence exposure from police, health, and child protection service records. Such information is vital for understanding the capabilities of government to identify those exposed to domestic and family violence and provide them with suitable services and early intervention. Second, future research relying on retrospective self-reports ought to prioritize the use of validated multi-item questionnaires, such as the Child Exposure to Domestic Violence Scale (Edleson et al., 2008) and Childhood Trauma Questionnaire (Bernstein et al., 1998), and corroborate reports from multiple respondents. Finally, more research from representative samples outside of North America is warranted to better evaluate cross-country differences in domestic and family violence exposure. Research should make additional efforts to include gender diverse individuals to ensure their appropriate representation in population-based findings.

## Conclusion

This review demonstrates that childhood exposure to physical domestic and family violence, including as a witness, is relatively common, affecting around one-in-six people by 18 years of age globally. There appears to be substantial regional variations in exposure, which may reflect differences in underlying economic conditions, cultural practices, and service availability. Nonetheless, the findings suggest that the child victims and witnesses of physical domestic and family violence should be given equal priority for intervention and prevention. However, additional population-based research, particularly from low- and middle-income countries, and disaggregated by sex, is needed to produce more reliable prevalence estimates that may guide policy and intervention efforts.

### Summary of Critical Findings

- Childhood exposure to physical domestic and family violence is relatively common, affecting around one-in-six people by 18 years of age globally
- A similar proportion of children were the victim or witness of physical domestic and family violence, although boys were slightly more likley than girls to be a victim.
- There were substantial regional variations in the prevalence of exposure, likely reflecting cross-country differences in economic conditions, cultural practices, and service availability.

### Implications for Practice, Policy, and Research

- More attention needs to be provided to children who witness physical domestic and family violence
- Lower-income countries may require further support for culturally sensitive domestic violence prevention and interventions services.
- More research is needed from studies using whole-of-population record linkage data, validated multi-item indicators, and samples from lower- and middle-income countries.

## Notes

1. Sample size calculation was based on an assumed 15% population prevalence of childhood exposure to physical domestic and family violence with a 5% margin of error and 99% confidence level (Naing et al., 2006).
